# Metabolic Perturbation Associated With COVID-19 Disease Severity and SARS-CoV-2 Replication

**DOI:** 10.1016/j.mcpro.2021.100159

**Published:** 2021-10-05

**Authors:** Shuba Krishnan, Hampus Nordqvist, Anoop T. Ambikan, Soham Gupta, Maike Sperk, Sara Svensson-Akusjärvi, Flora Mikaeloff, Rui Benfeitas, Elisa Saccon, Sivasankaran Munusamy Ponnan, Jimmy Esneider Rodriguez, Negin Nikouyan, Amani Odeh, Gustaf Ahlén, Muhammad Asghar, Matti Sällberg, Jan Vesterbacka, Piotr Nowak, Ákos Végvári, Anders Sönnerborg, Carl Johan Treutiger, Ujjwal Neogi

**Affiliations:** 1 Division of Clinical Microbiology, Department of Laboratory Medicine, Karolinska Institute, ANA Futura, Campus Flemingsberg, Stockholm, Sweden; 2 Södersjukhuset (The South General Hospital), Stockholm, Sweden; 3 National Bioinformatics Infrastructure Sweden (NBIS), Science for Life Laboratory, Department of Biochemistry and Biophysics, Stockholm University, Stockholm, Sweden; 4 Centre for Infectious Disease Research, Indian Institute of Science (IISc), Bangalore, Karnataka, India; 5 Division of Chemistry I, Department of Medical Biochemistry and Biophysics, Karolinska Institutet, Stockholm, Sweden; 6 Division of Infectious Diseases, Department of Medicine Solna, Karolinska Institutet, Stockholm, Sweden; 7 Department of Medicine Huddinge, Division of Infectious Diseases, Karolinska Institutet, Stockholm, Sweden; 8 The Laboratory for Molecular Infection Medicine Sweden MIMS, Umeå University, Umea, Sweden; 9 Manipal Institute of Virology (MIV), Manipal Academy of Higher Education, Manipal, Karnataka, India

**Keywords:** metabolomics, COVID-19, metabolic transporters, mannose, BMI, body mass index, COVID-19, coronavirus disease 2019, 2-DG, 2-deoxy-d-glucose, DON, 6-diazo-5-oxo-l-norleucine, ESI, electrospray ionization, GLUT, glucose transporter, HC, healthy control, HIF-1, hypoxia-inducible factor 1, IL, interleukin, IQR, interquartile range, MBL, mannose-binding lectin, MOI, multiplicity of infection, MS/MS, tandem mass spectroscopy, mtDNA, mitochondrial DNA, mTOR, mammalian target of rapamycin, O_2_, oxygen, PBMC, peripheral blood mononuclear cell, PPP, pentose phosphate pathway, RSD, relative standard deviation, SARS-CoV-2, severe acute respiratory syndrome coronavirus-2, TCA, tricarboxylic acid, UPLC, ultrahigh performance liquid chromatography

## Abstract

Viruses hijack host metabolic pathways for their replicative advantage. In this study, using patient-derived multiomics data and *in vitro* infection assays, we aimed to understand the role of key metabolic pathways that can regulate severe acute respiratory syndrome coronavirus-2 reproduction and their association with disease severity. We used multiomics platforms (targeted and untargeted proteomics and untargeted metabolomics) on patient samples and cell-line models along with immune phenotyping of metabolite transporters in patient blood cells to understand viral-induced metabolic modulations. We also modulated key metabolic pathways that were identified using multiomics data to regulate the viral reproduction *in vitro*. Coronavirus disease 2019 disease severity was characterized by increased plasma glucose and mannose levels. Immune phenotyping identified altered expression patterns of carbohydrate transporter, glucose transporter 1, in CD8^+^ T cells, intermediate and nonclassical monocytes, and amino acid transporter, xCT, in classical, intermediate, and nonclassical monocytes. In *in vitro* lung epithelial cell (Calu-3) infection model, we found that glycolysis and glutaminolysis are essential for virus replication, and blocking these metabolic pathways caused significant reduction in virus production. Taken together, we therefore hypothesized that severe acute respiratory syndrome coronavirus-2 utilizes and rewires pathways governing central carbon metabolism leading to the efflux of toxic metabolites and associated with disease severity. Thus, the host metabolic perturbation could be an attractive strategy to limit the viral replication and disease severity.

## Highlights

• COVID-19 disease severity was characterized by increased plasma glucose and mannose.
• Mannose is a strong biomarker of COVID-19 disease severity.
• Glycolysis and glutaminolysis are essential for virus replication.
• Blocking the metabolic pathways caused significant reduction in virus production.

## Graphical Abstract

**Figure:**
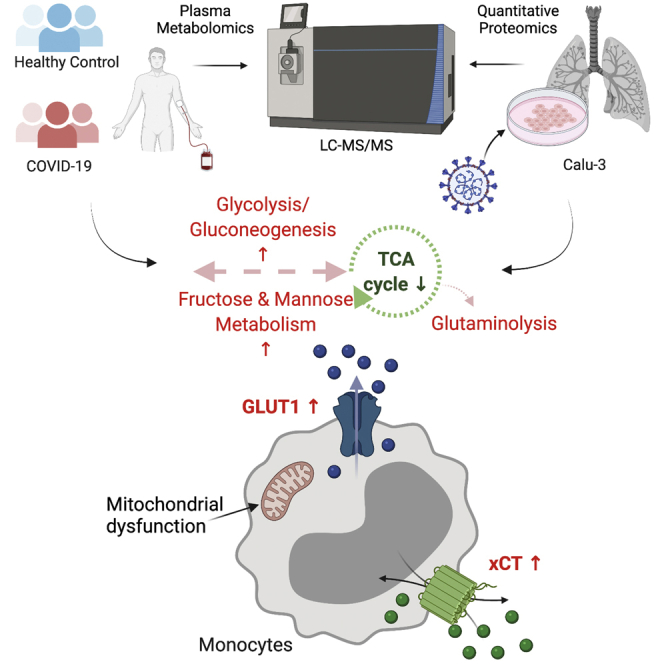

## In Brief

The molecular mechanisms underlying SARS-CoV-2 infection and disease severity are unclear. We used multiomics analysis in patient material and cell-line models to delineate the metabolic modulations caused by SARS-CoV-2 infection. Plasma mannose emerged as a strong biomarker for COVID-19 severity. SARS-CoV-2 depends on glutaminolysis and glycolysis for infection and replication. Infection of lung epithelial cells indicated strong metabolic adaptation with mitochondrial dysfunction. Inhibition of glycolysis and glutaminolysis limits viral production, which indicates a potential host-directed novel treatment strategy.

The global pandemic of coronavirus disease 2019 (COVID-19) caused by the severe acute respiratory syndrome coronavirus-2 (SARS-CoV-2) created a severe public health crisis worldwide. Although most patients presented with mild to moderate or no symptoms, patients having pre-existing metabolic disorders like diabetes, cardiovascular diseases, and obesity are at risk for severe and critical cases of infection. Some recent observational studies indicate that disease severity in patients with COVID-19 is associated with plasma metabolic abnormalities that include a shift toward amino acid and fatty acid synthesis, altered energy, and lipid metabolism (1, 2, 3, 4). However, metabolic regulation of an individual always depends on several factors, including age, gender, environmental factors, dietary intake, and lifestyle. Such alterations in metabolic regulation can change rapidly or adapt to an altered situation, and sometimes have sustained effects over an extended period. The initial phase of characterization of the metabolic landscape of COVID-19 and its association with disease severity has urged the need to understand how metabolic reprogramming occurs during the acute SARS-CoV-2 infection with the ultimate goal toward therapeutic intervention.

Viruses are known to exploit the host metabolic machinery to meet their biosynthetic demands for optimal replication capacity (5). This cellular exploration is highly connected with the initial host–viral response, thereby determining the disease pathogenesis. Viral replication is dependent on extracellular carbon sources such as glucose and glutamine. It induces a plethora of metabolic alterations in host cell including host central carbon metabolism, nucleotide, fatty acids, and lipid synthesis that modulate viral pathogenesis and host response (6). Our recent *in vitro* multiomics studies have shown that the SARS-CoV-2 dysregulates PI3K/Akt/mammalian target of rapamycin (mTOR) and hypoxia-inducible factor 1 (HIF-1) signaling in infected cells (7). These pathways regulate glycolysis by altering glucose transporters (GLUTs) across cell membranes. Targeting these pathways with inhibitors such as MK2206 (Akt inhibitor) or 2-deoxy-d-glucose (2-DG; glycolysis inhibitor) can lower the viral burden in the cells *in vitro* (7, 8). This opens the area for host-based metabolic strategies to inhibit viruses as an additional way other than direct-acting antivirals to weaken the viral replication by metabolic intervention.

In this study, we performed plasma proteomics targeting 92 plasma proteins related to inflammation and plasma untargeted metabolomics followed by immune phenotyping of the lymphocyte and monocyte cell populations toward the metabolite transporters. To understand the viral-induced metabolic rewiring, we reanalyzed quantitative untargeted proteomics data in SARS-CoV-2–infected lung, liver, kidney, and colon-derived cell lines (9). We also modulated the key metabolic pathways identified in the patient-based metabolomics data and cell model–based quantitative proteomics data to regulate the viral reproduction. Our clinical and experimental studies thus provide an account of metabolic control during SARS-CoV-2 infection that can aid antiviral therapeutics in COVID-19 through metabolic perturbation.

## Experimental Procedures

### Study Designing and Patients

The COVID-19 patients (n = 41) who were PCR positive and hospitalized in May 2020 were recruited from the South Hospital, Stockholm. Based on the oxygen (O_2_) requirements, the patients were categorized into (1) hospitalized—mild (O_2_ consumption <4 l/min) and (2) hospitalized—severe (O_2_ consumption ≥4 l/min). The exclusion criteria included known liver cirrhosis, severe renal insufficiency (estimated glomerular filtration rate ≤30 ml/min/1.73 m^2^), chronic obstructive pulmonary disease, and chronic lung disease leading to habitual SpO_2_ ≤ 92%. In addition, COVID-19 PCR-negative samples (healthy control [HC], n = 31) were also collected. Immunoglobulin G antibody was tested on the HC samples as described previously (10), and ten samples turned out to be SARS-CoV-2 Ab positive further defined as HC-CoV-2 Ab+. The study was approved by regional ethics committees of Stockholm (dnr 2020-01865) and abide by the Declaration of Helsinki principles. All participants gave informed consent. The patient identity was anonymized and delinked before analysis.

### Cell Line and Virus

Human lung adenocarcinoma cell line, Calu-3 (American Type Culture Collection; HTB-55), was purchased. SARS-CoV-2 virus used in this study was the first virus isolated from a Swedish patient (11).

### Chemicals and Materials

All information regarding reagents, antibodies, and critical commercial kits are listed in Table S1.

### Experimental Design and Statistical Rationale

Plasma untargeted metabolomics was performed by ultrahigh performance LC–tandem mass spectroscopy (UPLC-MS/MS) using global metabolomics (HD4) in Metabolon, Inc as described by us recently (12). The samples were run in four different UPLC–MS/MS methods: two separate reverse phase/UPLC-MS/MS methods with positive ion mode electrospray ionization (ESI), one for analysis by reverse phase/UPLC–MS/MS with negative ion mode ESI, and one for analysis by hydrophilic interaction LC/UPLC–MS/MS with negative ion mode ESI, with a single analysis for each sample (no technical or biological replicates). Several types of controls were analyzed in concert with the experimental samples: a pooled well-characterized human plasma maintained by Metabolon, Inc served as a technical replicate throughout the dataset and injected as every eighth samples; extracted water samples served as process blanks injected as every fourth, and a cocktail of quality control standards that were carefully chosen not to interfere with the measurement of endogenous compounds were spiked into every analyzed sample, allowed instrument performance monitoring, and aided chromatographic alignment. Instrument variability was determined by calculating the median relative standard deviation (RSD) for the internal standards that were added to each sample prior to injection into the mass spectrometers. The median RSD for the instrument variability of the run was 7%. Overall process variability was determined by calculating the median RSD for all endogenous metabolites (*i.e.*, noninstrument standards) present in the pooled quality control technical replicates, which was 9%. The samples were randomized of the individuals from the study design groups. The metabolomics method is ISO 9001:2015 certified, and the laboratory is accredited by the College of American Pathologist, USA. For statistical analysis, we sought to explore the effect of COVID-19 on the measured plasma metabolites. The model also contained potential confounders of the plasma metabolic profile, that is, age, gender, and body mass index (BMI). For the cellular proteomics, two experimental groups were mocked, and SARS-CoV-2 was infected for all the four cell lines, Caco-2, Calu-3 (American Type Culture Collection; HTB-55), Huh7, and 293FT as reported earlier (9). The LC–MS/MS assay was carried out in three technical replicates. All the mock and infected cells were performed in the same batch to minimize the issue of the typical missing values (13) and avoid batch effects. All reported *p* values were corrected (Benjamini–Hochberg) throughout and considered statistically significant if <0.05 unless otherwise stated.

### Plasma Targeted Proteomics Analyses (Secretome)

The secretome was performed using a targeted proteomics analysis by proximity extension assay technology targeting 96 plasma protein markers by Olink Target 96 Immuno-Oncology (Olink). The protein concentration was reported as normalized protein expression levels (NPX), which were Ct values normalized to extension control and a correction factor (normal background noise) and reported in Log2 scale.

### Statistical and Bioinformatics Analysis

For targeted proteomics data analysis, we used Mann–Whitney *U* test through the R package stats, version 3.6.1, for pair-wise analysis as the data were not normally distributed. For metabolomics data, dimensionality reduction of all samples was performed with uniform manifold approximation and projection (UMAP) using R package UMAP, version 0.2.6.0 (14). Reduced dimensions of the data were plotted in 2D space using R package ggplot, version 3.3.2 (15). The metabolite measurements were log2 scaled before differential analysis. Differential analysis was done using R/Bioconductor package limma, version 3.42.2 (16). R package MUVR, version 0.0.973 (17), was used for biomarker discovery. Random forest core modeling was selected from the package for biomarker identification. Minimal-optimal variables selected by the model were considered as biomarkers. Correlation analysis was performed using corr.test function from the package psych, version 1.9.12.31, based on Spearman rank correlations. Untargeted protein raw data abundance was first filtered for empty rows and quantile normalized. Functional analysis of the proteins was performed using enrichr module of python package GSEAPY, version 0.9.16 (https://pypi.org/project/gseapy/) (18), where all the quantified proteins were considered as background. Kyoto Encyclopedia of Genes and Genomes 2019 human gene-set library downloaded from Enrichr web resources was used for the enrichment test for molecular pathway analysis. Functional analysis of the metabolites was carried out using ingenuity pathway analysis software package.

### Data Visualization

Heatmaps were generated using R/Bioconductor package ComplexHeatmap, version 2.2.0 (19). Violin plots, box plots, bubble plots, and volcano plots were made using R package ggplot2, version 3.3.2, respectively. A density plot was created using the R package ggridges, version 0.5.2. Alluvial plot was made using the R package ggalluvial, version 0.11.3. Correlation pairs plot was made using R package GGally, version 2.0.0. The network was represented using Cytoscape, version 3.6.1 (https://cytoscape.org/). Protein–protein interactions were retrieved from STRING Db (version 5.0) (https://string-db.org/). Only interactions with high confidence (interaction score > 0.7) from databases and experiences were kept.

### Flow Cytometry

Peripheral blood mononuclear cells (PBMCs) were subjected to flow cytometry analysis. All samples were stained with live/dead fixable near IR dye (Invitrogen), and cell surface markers were detected by incubating cells with relevant antibodies for 20 min on ice in flow cytometry buffer. All cells were fixed with 2% paraformaldehyde before acquiring a BD FACS Symphony flow cytometer (BD Bioscience), and data were analyzed and compensated with FlowJo, version 10.6.2 (TreeStar, Inc) and Prism 8 (GraphPad Software, Inc). The gating strategy is given in supplemental Figure S1.

### Measurement of Mitochondrial DNA Copy Number

Mitochondrial DNA (mtDNA) copy number was measured using an absolute human telomere length and Mitochondrial DNA Copy Number Dual Quantification qPCR Assay Kit (ScienCell Research Laboratories) as per the manufacturer's instruction. A reference genomic DNA was added on each plate with known mtDNA copy number (925 copies). Each sample was run in duplicates, and relative mtDNA copy number and single copy reference were calculated by ΔCT (CT target sample − CT reference sample), after adjusting PCR efficiency using the Pfaffl method (20). Finally, mtDNA copy number per diploid cell of target sample to reference sample was calculated by (2−^ΔΔCT^ × 925), where ΔΔCT is ΔCT mtDNA/ΔCT single copy reference.

### SARS-CoV-2 Infection and Quantitative Proteomics

To identify the acute SARS-CoV-2–induced metabolic responses, we reanalyzed our previous untargeted quantitative proteomics data on SARS-CoV-2–infected human cell lines, including Calu-3 (lung), Caco-2 (colon), 293FT (kidney), and Huh7 (liver) after 24 h of infection restricted to metabolic pathways (9).

### Metabolic Perturbation and Virus Infection

Calu-3 cells were seeded in 24-well plate, and after 72 h of seeding, the cells were infected with SARS-CoV-2 at multiplicity of infection (MOI) of 0.001 for 1 h. Following infection, the cells were treated with Dulbecco's modified Eagle's medium (Gibco), which contained pyruvate (1 mM) and glutamine (4 mM) as the basal carbon source and were supplemented with 5% fetal bovine serum and different concentrations of glucose (11.1, 22.2, and 44.4 mM) (Gibco) and keeping basal glucose concentration at 11.1 mM, different concentrations of mannose (11.1, 22.2, and 44.4 mM) (Sigma–Aldrich). Inhibitors of glycolysis, 2-DG (Sigma–Aldrich), and glutaminolysis, 6-diazo-5-oxo-l-norleucine (DON; Sigma–Aldrich), were reconstituted in water, and cytotoxicity at different concentrations and 24 h time point was determined in Calu-3 cells using alamarBlue Cell Viability Reagent (Invitrogen) according to the manufacturer's instructions. To determine the effect of these drugs on viral replication, following 1 hpi (MOI = 0.001), the Calu-3 cells were treated with 2-DG (10 mM) and DON (200 μM), respectively. The supernatants were collected after 24 hpi, and the cells were lysed in TRI reagent (Zymo Research) and stored in −70 °C for RNA extraction.

### RT–Quantitative PCR Analysis

The virus production and infectivity were determined by RT–quantitative PCR targeting the viral *E-gene* in the supernatant and RNA extracted from the cells as described by us (7, 9).

The purified RNA was reverse transcribed using a High-Capacity cDNA reverse transcription kit (Applied Biosystems) according to the manufacturer's instructions. Quantitative PCRs were performed using KAPA SYBR Fast qPCR kit (KAPA Biosystems) on an Applied Biosystems 7500 Fast qPCR machine. Detailed information on primers is included in supplemental Table S2.

### Plasma Mannose-Binding Lectin Measurement

Mannose-binding lectin (MBL) levels in patient plasma were determined using Human MBL Quantikine ELISA Kit (R&D Systems) according to the manufacturer's instructions using NanoQuant Infinite M200 plate reader (Tecan).

## Results

### Patient Characteristics

The study population included HCs (n = 31), SARS-CoV-2 PCR-positive hospitalized—mild (mild, O_2_ consumption <4 l/min, n = 29) and hospitalized—severe (severe, O_2_ consumption ≥4 l/min, n = 12) patients. The mild and severe groups were matched by gender (male: 79% *versus* 91%, *p* = 0.6514), BMI (median [interquartile range (IQR)]: 29 (25–31) *versus* 28 (25–34); *p* = 0.8622), and age (median [IQR]: 57 [44–63] *versus* 57 [52–69]; *p* = 0.2831). The HC has significantly lower age (median [IQR]: 48 (46–55)) and lower BMI (median [IQR]: 24 (21–25)) (supplemental Table S3). The immunoglobulin G CoV-2 antibody test showed that ten of the HC were CoV-2 antibody positive (HC-CoV-2 Ab+; supplemental Fig. S2). Among the patients with COVID-19, the classical comorbidities were observed at 45% (13 of 29) in mild and at 66% (8 of 12) in severe (*p* = 0.3058). The samples were collected within median (IQR) 2 (2–3.5) days of hospitalization (median [IQR] mild: 2 (1–3) and severe 3 (2–4); *p* = 0.1170). None of the patients were on any known anti-COVID-19 treatment at the time of sample collection.

### Plasma Proteomics Identified Distinct Clusters of HC and COVID-19 Individuals

**Fig. 1:**
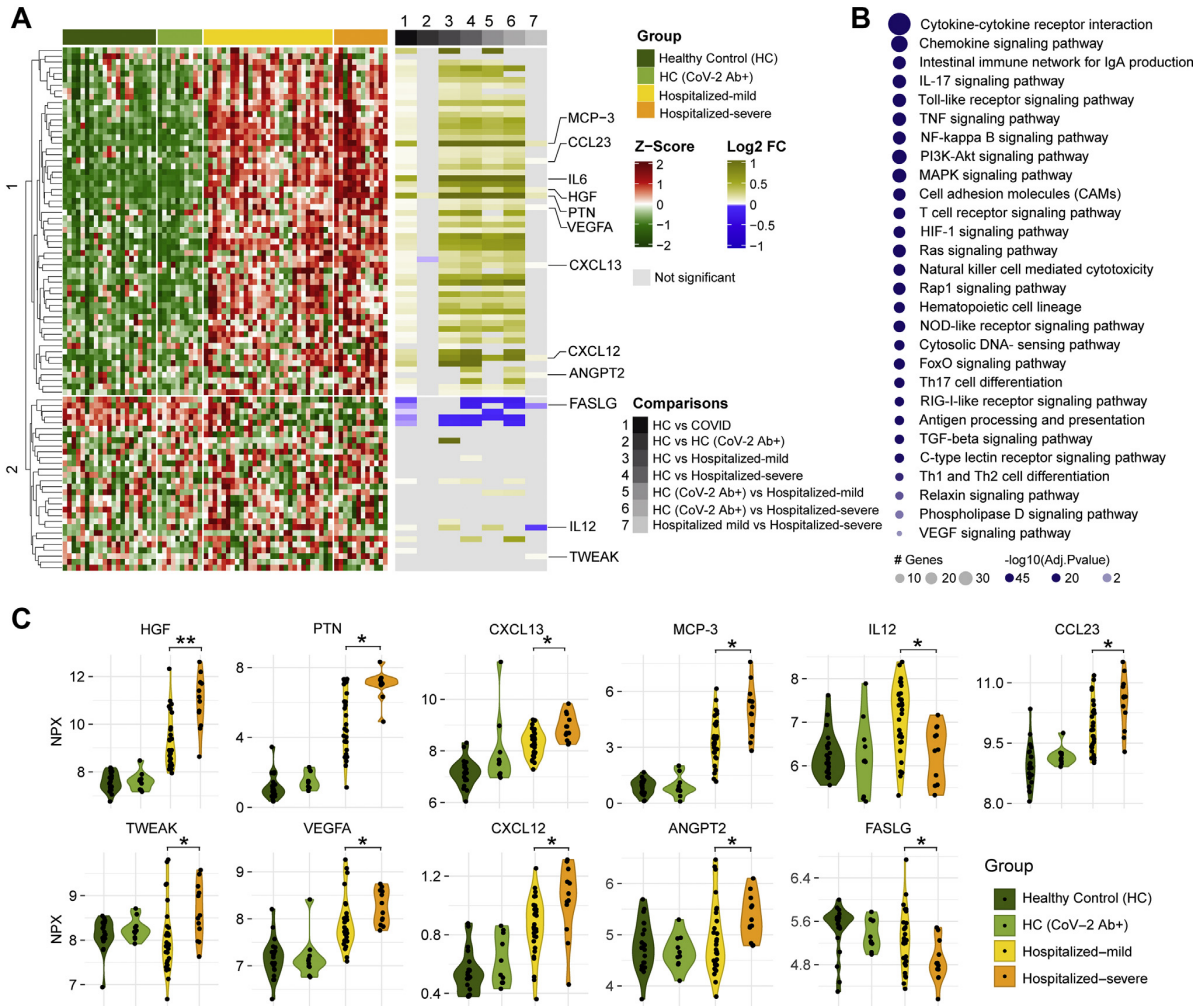
**Targeted plasma proteomics in COVID-19 patients.***A*, heatmap of Z-score transformed quantitative measurements of all proteins detected by the immuno-oncology panel. Column annotation represents each patient sample and their corresponding groups and pairs of statistical analysis. Rows are proteins hierarchically clustered based on the Euclidean distance and complete linkage method. Names of proteins that are identified as significant in any of the statistical analysis are printed. *B*, KEGG pathway enrichment analysis results of significantly changed proteins between HC (HC + HC-CoV-2 Ab+) and COVID-19 (hospitalized mild + hospitalized severe) groups. *C*, violin plot of significantly regulated (Mann–Whitney *U* test) proteins between hospitalized mild and hospitalized severe, ∗adjusted *p* < 0.05, ∗∗adjusted *p* < 0.01. COVID-19, coronavirus disease 2019; HC, healthy control; KEGG, Kyoto Encyclopedia of Genes and Genomes.

We performed targeted proteomics analyses (secretome) looking at 92 plasma proteins involved in inflammatory responses. As expected, several cytokines and chemokines were significantly elevated in COVID-19 patients (mild and severe) compared with HCs (HC and HC-CoV-2 Ab+), including interleukin 6 (IL-6) (Fig. 1*A*). Pathway enrichment analyses of the proteins that were significantly changed between HCs and COVID-19 patients revealed that the majority of altered proteins were involved in cytokine–cytokine receptor interaction and chemokine signaling, followed by intestinal network for immunoglobulin A production, IL-17 signaling pathway, and Toll-like receptor signaling pathway; to name the top five pathways (Fig. 1*B*). Interestingly, 11 proteins were altered between the mild and severe COVID-19 patients (Fig. 1, *A* and *C*): hepatocyte growth factor, pleiotrophin, the chemokines CXC chemokine ligand 12, CXC chemokine ligand 13, and chemokine ligand 23 (also known as macrophage inflammatory protein 3), monocyte chemotactic protein 3 (also known as chemokine ligand 7), IL-12, tumor necrosis factor–like weak inducer of apoptosis, vascular endothelial growth factor A, angiopoietin 2, and Fas ligand (adjusted *p* < 0.05). Most of these proteins were elevated in COVID-19 patients with highest levels in the severe group, except for IL-12, which was increased in the mild group compared with both severe group and HCs (Fig. 1, *A* and *B*). Furthermore, Fas ligand followed the opposite trend of being lower in COVID-19 patients compared with HC and lowest in severe COVID-19 patients.

### Distinct Amino Acid and Carbohydrate Profile in COVID-19 Patients

**Fig. 2:**
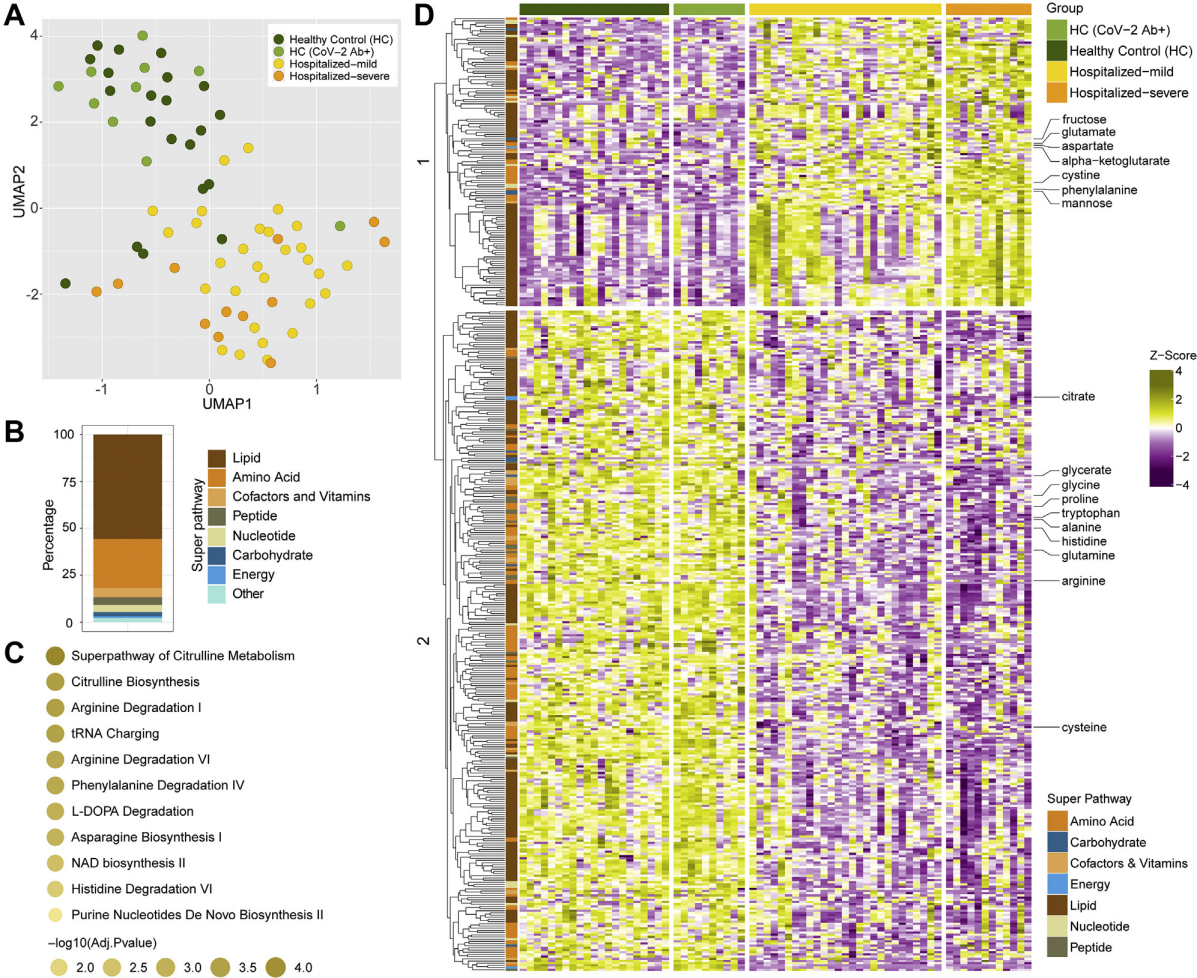
**Untargeted global plasma metabolomics in COVID-19 patients.***A*, sample distribution for quantitative metabolite measurements plotted in 2D space after performing dimensionality reduction using UMAP. *B*, stacked bar plots visualizing percentage of metabolites significantly changed between HC (HC + HC-CoV-2 Ab+) and COVID-19 (hospitalized mild + hospitalized severe) group concerning their corresponding superpathways and subpathways. *C*, metabolic set enrichment analysis using the significantly enriched metabolites between HCs and COVID-19 patients. The size of the bubble indicates adjusted *p* values. *D*, heatmap of log2 scaled and Z-score transformed measurements of metabolites significantly changed between HC (HC + HC-CoV-2 Ab+) and COVID-19 (hospitalized mild + hospitalized severe) groups. Column annotation represents each patient sample and the corresponding groups. Row annotation represents superpathways of the metabolites. Rows are metabolites hierarchically clustered based on Euclidean distance and complete linkage method. COVID-19, coronavirus disease 2019; HC, healthy control; UMAP, uniform manifold approximation and projection.

The plasma metabolomic profile followed a pattern similar to the plasma proteomics. However, no metabolites were significantly different between HC and HC-CoV-2 Ab+ (adjusted *p* > 0.05). Therefore, we combined the two groups as HC for further analysis. The distribution of all samples for metabolite enrichment showed a fair separation between samples of HC and COVID-19 patients (Fig. 2*A*). Differential analysis between COVID-19 patients and healthy individuals after adjusting for age, gender, and BMI identified 444 significantly regulated metabolites (adjusted *p* < 0.05), many of which are lipids followed by amino acids (Fig. 2*B*). Metabolite set enrichment analysis of the significant metabolites (adjusted *p* < 0.05) identified amino acid–related pathways were most predominantly affected during infection, as shown in Figure 2*C*. Hierarchical clustering of the metabolites showed two clusters that had distinct enrichment patterns in COVID-19 infected patients compared with HCs (Fig. 2*D*). Among these, amino acids such as glycine, proline, tryptophan, alanine, histidine, glutamine, and arginine, were found in lower levels in COVID-19 patients, whereas glutamate, aspartate, and phenylalanine were found in higher levels (Fig. 2*D* and supplemental Fig. S3) as also observed in earlier studies (1, 3, 21). Interestingly, metabolites of the central carbon metabolism including glycolysis (glucose, 3-phosphoglycerate, pyruvate, and lactate) and tricarboxylic acid (TCA) cycle (citrate, aconitate, and α-ketoglutarate) showed distinct changes when comparing HCs and COVID-19 patients and the different COVID-19 disease states (supplemental Fig. S4).

### Severe COVID-19 Patients Show a Distinct Metabolic Profile With Mannose as a Biomarker

**Fig. 3:**
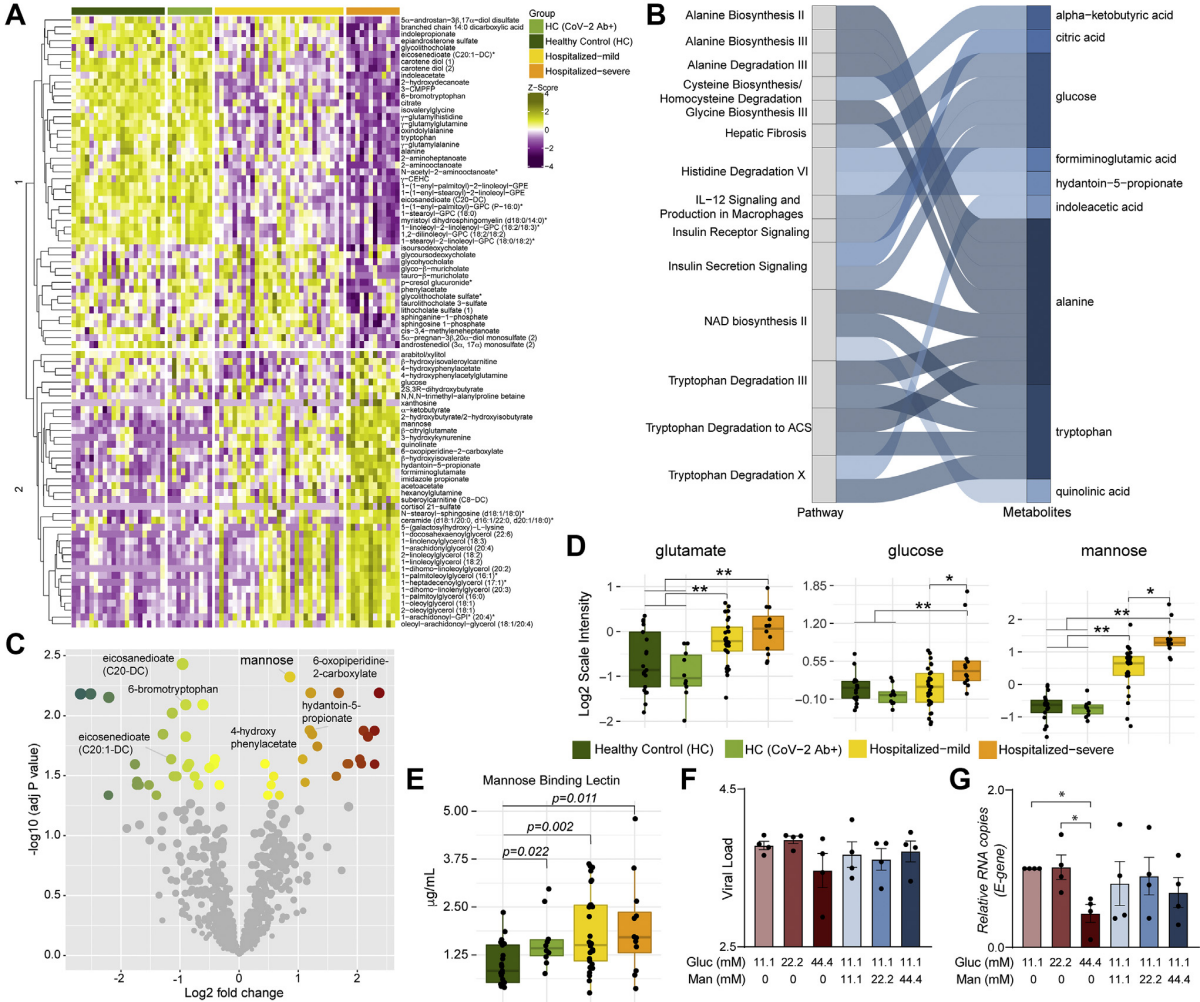
**Metabolic alterations in the mild and severe hospitalized COVID-19 patients and its role in viral replication.***A*, heatmap of log2 scaled and Z-score transformed significantly changed metabolites between hospitalized mild and hospitalized severe groups. Column annotation represents each patient sample and the corresponding groups. Rows are metabolites hierarchically clustered based on Euclidean distance and complete linkage method. *B*, alluvial plot representing pathways resulted from IPA pathway enrichment analysis using all metabolites that differ significantly between hospitalized mild and severe groups. *C*, volcano plot showing all the metabolites that differ significantly between hospitalized mild and hospitalized severe groups. *D*, box plots of key metabolites glutamate, glucose, and mannose. Adjusted *p* values determined by limma is shown. ∗adjusted *p* < 0.05 and ∗∗adjusted *p* < 0.001. *E*, box plots of soluble mannose-binding lectin levels in patients' plasma. *p* Values determined by Mann–Whitney *U* test. *F* and *G*, viral load of SARS-CoV-2 determined by RT–quantitative PCR targeting the viral *E-gene* is measured in (*F*) cell culture supernatants and (*G*) cell lysates at MOI 0.001 in Calu-3 cells grown in different glucose and mannose concentrations (millimolar) as indicated. The data are represented as mean ± SEM of two independent experiments, duplicates in each experiment. *p* Values are determined by Student's *t* test, ∗*p* < 0.05. COVID-19, coronavirus disease 2019; IPA, ingenuity pathway analysis; MOI, multiplicity of infection; SARS-CoV-2, severe acute respiratory syndrome coronavirus-2.

Next, we aimed to identify the metabolic signature in COVID-19 severe patients. Statistical analysis found 88 metabolites that significantly differed in the severe group compared with mild samples. Hierarchical clustering of the metabolites showed two clusters with moderately distinct enrichment patterns in severe samples compared with mild samples (Fig. 3*A*). Metabolic pathway enrichment analysis of the significant metabolites by ingenuity pathway analysis showed that several amino acid–related pathways, IL-12 signaling, and production in macrophages and insulin signaling pathways were mainly dysregulated in the severe patient samples compared with mild ones (Fig. 3*B*). Next, we sought to identify biomarkers that differentiate the severe and mild samples. Using R package MUVR that is suitable for small sample size, a total of eight metabolites were identified as biomarkers (supplemental Fig. S5). After adjusting for age, gender, and BMI, seven remained significant, of which four had higher abundance and three had lower abundance in COVID-19 severe patients compared with the mild ones (Fig. 3*C* and supplemental Fig. S5). COVID-19 infection was associated with increased glutamate levels, whereas glucose and mannose were determinants of the severity of the disease (Fig. 3*D*) and mannose can be considered as a biomarker for disease severity. These data suggest alterations in the glycolysis/gluconeogenesis, glutaminolysis, and mannose metabolism in COVID-19 patients irrespective of severity. Our targeted secretome data identified the C-type lectin receptor signaling pathway as one of the top 28 ranked protein pathways that were significantly changed between HCs and COVID-19 patients (Fig. 1*B*). MBLs are involved in complement activation through the lectin pathways and have the ability to cause increased coagulopathy, a feature often associated with COVID-19 severity (22). We, therefore, measured plasma levels of soluble MBL using ELISA and observed an increase in COVID-19 patients compared with HC. Strikingly, there was no significant difference between the mild and severe COVID-19 patients, but all COVID-19 patients and the HC-CoV-2 Ab+ individuals showed increased MBL levels compared with HC (Fig. 3*E*). These data show a prominent elevation of MBL during COVID-19 infection that can persist over a prolonged duration of time after recovery. MBLs play an important role in viral pathogenesis as they recognize the glycans present in the viral envelope and subsequently activating antiviral immune response and T cells (23, 24). While the role of circulating mannose levels on activation of MBL-mediated immune response is not known, we assessed the correlation between them. We did not observe any correlation between MBL and mannose in COVID-19 patients (Spearman correlation: 0.1437 (95% confidence interval: −0.1806 to 0.4399]). We, therefore, speculate that elevated MBL might not directly be the consequence of the higher plasma mannose levels but regulated by SARS-CoV-2.

### Role of Increased Sugars in SARS-CoV-2 Infection *In vitro*

An earlier study reported an increased SARS-CoV-2 replication in monocytes in the presence of elevated glucose (25). However, a more recent study showed that despite SARS-CoV-2 efficiently infects monocytes and monocyte-derived macrophages, their infection is abortive (26). Therefore, to understand the role of sugars like glucose and mannose in SARS-CoV-2 infection, we performed *in vitro* infection assays in Calu-3 cells with varying media concentrations of glucose (11.1, 22.2, and 44.4 mM) and mannose (0, 11.1, 22.2, and 44.4 mM) with 0.001 MOI. We did not observe any statistically significant difference in virus production in the supernatant in the glucose/mannose concentrations tested (*p* > 0.05; Fig. 3*F*), whereas we found a significant reduction in viral *E-gene* expression at cellular level with high glucose concentration of 44.4 mM (Fig. 3*G*; *p* < 0.05). Supplementation with high mannose did not cause any significant change in expression of the *E-gene*. Overall, our data indicate that increased glucose levels but not mannose levels influence viral replication *in vitro* in Calu-3 cells.

### Immune Phenotyping of Metabolite Transporters GLUT1 (SLC2A1) and xCT (SLC7A11) in PBMCs

**Fig. 4:**
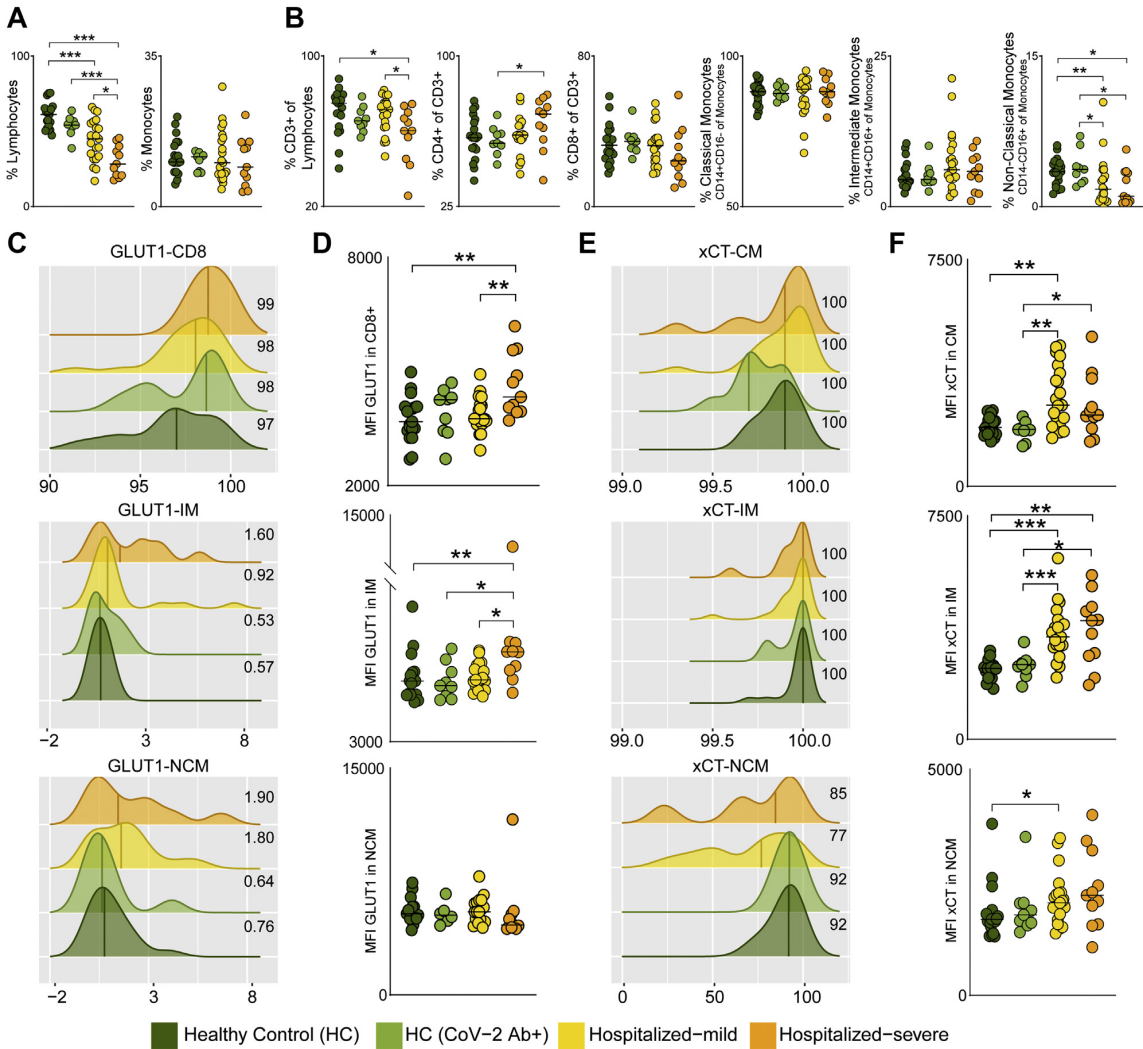
**Glucose, mannose, and glutamate transporters in COVID-19 severity.***A*, percentage of total lymphocytes and monocytes in all four patient groups. *B*, percentage of PBMC subpopulations, CD3^+^ T cells of lymphocytes, CD4^+^ T cells (of CD3^+^ cells), CD8^+^ T cells (of CD3^+^ cells), classical monocytes (CM, CD14^+^CD16^−^ of monocytes), intermediate monocytes (IM, CD14^+^CD16^+^ of monocytes), and nonclassical monocytes (NCM, CD14^−^CD16^+^ of monocytes). Median values are indicated by lines. *C*, density plot of percentage of CD8^+^ T cells, IM, and NCM expressing GLUT1. Histograms show percentage of cells expressing GLUT1 (*x*-axis) and GLUT1 read density of each sample (*y*-axis). The median percentage of cells expressing GLUT1 is shown for each patient group. *D*, MFI of GLUT1 in CD8^+^ T cells, IM and NCM in all four patient groups. *E*, density plot of percentage of CM, IM, and NCM expressing xCT. Histograms show percentage of cells expressing xCT (*x*-axis) and xCT read density of each sample (*y*-axis). The median percentage of cells expressing xCT is shown for each patient group. *F*, MFI of xCT in CM, IM, and NCM in all four patient groups. In all the panels, the median values are indicated by lines, *p* values are determined by Mann–Whitney *U* test, ∗*p* < 0.05, ∗∗*p* < 0.01, and ∗∗∗*p* < 0.001. COVID-19, coronavirus disease 2019; GLUT1, glucose transporter 1; MFI, mean fluorescence intensity; PBMC, peripheral blood mononuclear cell.

Metabolite transporters are known to dictate immune cell activity by controlling access to nutrients, thereby maintaining cellular homeostasis (27). Therefore, we next measured the expression of transporters, GLUT1 (SLC2A1) and xCT (SLC7A11), that are key transporters of glucose/mannose and antiporter of glutamate respectively, in PBMCs of HC (n = 19), HC-CoV-2 Ab+ (n = 9), and COVID-19 patients: mild (n = 21) and severe (n = 11) using flow cytometry. Like other studies (28, 29), the relative frequency of lymphocytes significantly decreased in COVID-19 patients compared to HCs, which was more prominent in severe patients (Fig. 4*A*). In total lymphocytic populations, the CD3^+^ T cells were significantly reduced in COVID-19 severe patients compared with mild patients and HCs (Fig. 4*B*). Although there was no difference in total monocyte frequencies, we observed a mild increase in the frequency of intermediate monocytes and a significant decrease in nonclassical monocytes in the COVID-19 patients compared to HCs (Fig. 4, *A* and *B*). This highlights the potential role of monocytes in COVID-19 as was also recently reported in single-cell transcriptomics data (4, 30) and functional analysis on COVID-19 patient monocytes (31). More than 98% CD8^+^ T cells expressed GLUT1, and the surface expression of GLUT1 was significantly higher in COVID-19 severe patients than in COVID-19 mild and HCs (Fig. 4, *C* and *D*). We also found significantly higher surface expression of GLUT1 on the intermediate monocytes of COVID-19 severe patients compared with COVID-19 mild and HCs (Fig. 4*D*). While all classical and intermediate monocytes expressed xCT, we observed a mild decrease in frequency of nonclassical monocytes expressing xCT in COVID-19 patients compared with HCs (Fig. 4*E*). The surface expression of xCT was significantly higher in classical and intermediate monocytes of COVID-19 patients compared to HCs (Fig. 4*F*), emphasizing the potential role of metabolite transporters in monocytes in COVID-19 infection.

### SARS-CoV-2 Regulates Central Carbon Metabolic Pathways in a Cell Type–Specific Manner

**Fig. 5:**
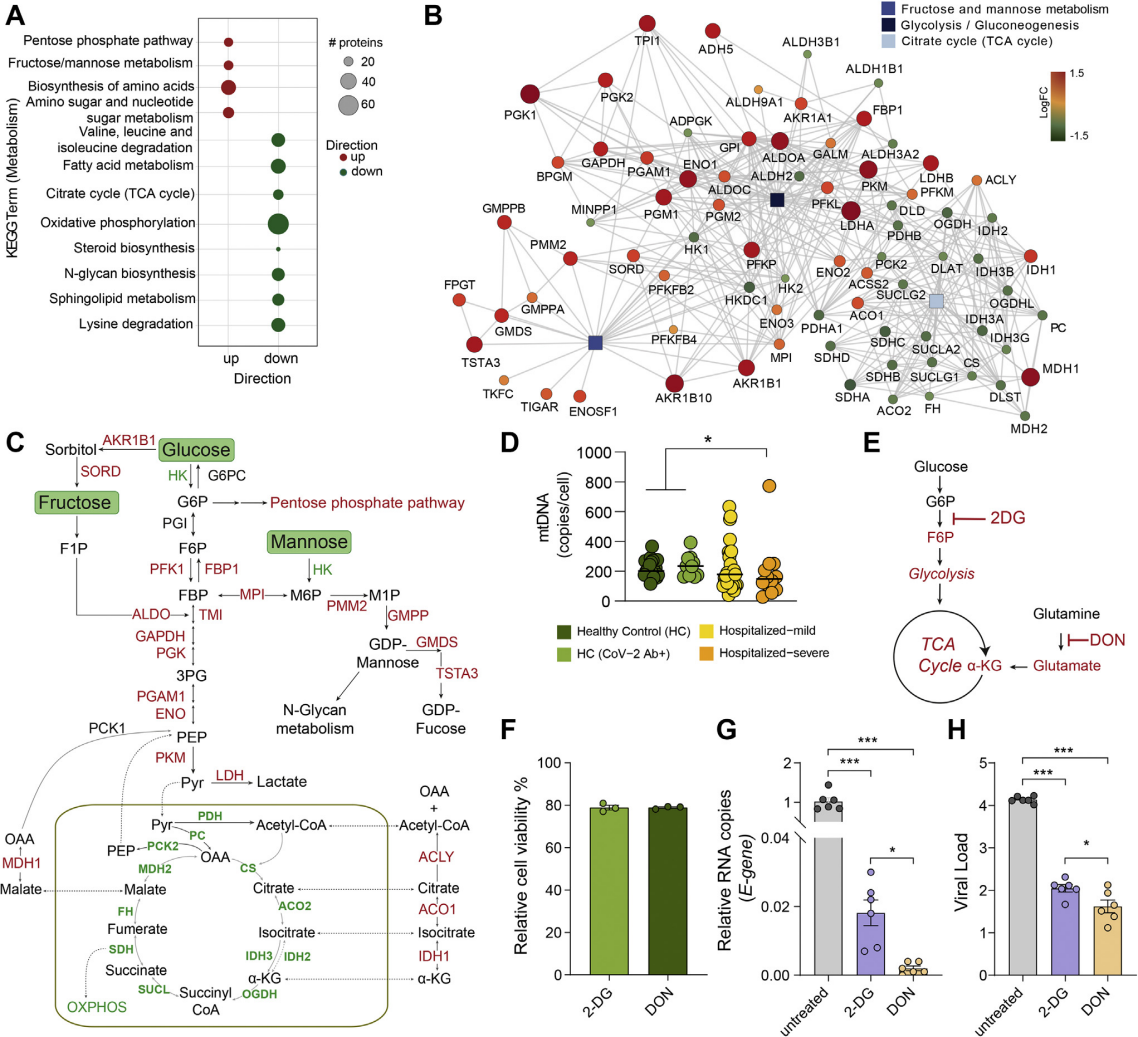
**Cell-specific regulation of central carbon metabolic pathways by SARS-CoV-2.***A*, bubble plots of protein set enrichment analysis (adjusted *p* < 0.1) restricted to metabolic pathways showing highly upregulated (*red*) and downregulated (*green*) proteins in SARS-CoV-2–infected Calu-3 cells compared with mock-infected cells. Bubble size is relative to number of proteins. *B*, network analysis of proteins from glycolysis/gluconeogenesis, fructose and mannose metabolism, and TCA cycle that were significantly different in SARS-CoV-2–infected and mock-infected Calu-3 cells. Rectangular shapes represent the three pathways. Circular shapes show each protein that is either upregulated (*red*) or downregulated (*green*) in infected cells compared with mock-infected cells. The size of the *circle* indicates fold change. *Lines* denote connection of each protein to its respective pathway and connection between each protein–protein (STRING, confidence > 0.7). *C*, schematic map of the glycolysis/gluconeogenesis, fructose and mannose metabolism, and TCA cycle. *Red* indicates significantly upregulated proteins, and *green* indicates significantly downregulated proteins in SARS-CoV-2–infected Calu-3 cells. *D*, mtDNA copy number in whole blood cells in all four patient groups. Median values are indicated by lines, *p* values are determined by Mann–Whitney *U* test, ∗*p* < 0.05. *E*, schematic of inhibitors of metabolic pathways, 2-DG inhibits glycolysis, and DON inhibits glutaminolysis. *F*, cell viability (percent relative to control) following treatment of Calu-3 cells with 2-DG (10 mM) and DON (200 μM). *G* and *H*, viral load of SARS-CoV-2 determined by RT–quantitative PCR targeting the viral *E-gene* is measured in (*G*) cell lysates and (*H*) cell culture supernatants, at MOI 0.001 in Calu-3 cells treated with 2-DG or DON as indicated. The data are represented as mean ± SEM of two individual experiments, triplicates in each experiment. *p* Values are determined by Student's *t* test, ∗*p* < 0.05, ∗∗∗*p* < 0.001. 2-DG, 2-deoxy-d-glucose; DON, 6-diazo-5-oxo-l-norleucine; MOI, multiplicity of infection; mtDNA, mitochondrial DNA; SARS-CoV-2, severe acute respiratory syndrome coronavirus-2; TCA, tricarboxylic acid.

Our previous study, together with other observational studies, indicate that SARS-CoV-2 infection causes dysregulation of PI3K/Akt/mTOR and HIF-1 signaling pathways (7, 25, 32) and affect mitochondrial functions (31, 33). Based on these findings, we hypothesized that the altered extracellular glucose, mannose, and glutamate levels could be due to dysregulated carbohydrate metabolism and mitochondrial function. Therefore, to identify the acute SARS-CoV-2–induced metabolic responses, we used our previous untargeted quantitative proteomics data on SARS-CoV-2–infected human cell lines, including Calu-3 (lung), Caco-2 (colon), 293FT (kidney), and Huh7 (liver) that showed differential protein abundance in Calu-3 (6462 proteins), Caco-2 (177 proteins), and Huh7 (4 proteins) after 24 h of infection (9). The protein set enrichment analysis targeting the metabolic pathways identified that most of the highly abundant proteins in infected Calu-3 cells belonged to pentose phosphate pathway (PPP), fructose and mannose metabolism, as well as amino acid biosynthesis (Fig. 5*A*). Proteins detected at a lower level in the infected cells mainly belonged to TCA cycle, oxidative phosphorylation, and N-glycan biosynthesis (Fig. 5*A*). Parallelly, in patients' metabolomic analysis, we observed unbalanced levels of glycolysis, fructose and mannose metabolism, and TCA cycle intermediates (Figs. 2*D* and 3*D*). We, therefore, focused our analysis on the proteins (n = 78) that are a part of glycolysis/gluconeogenesis, fructose and mannose metabolism, and TCA cycle (Kyoto Encyclopedia of Genes and Genomes Human 2019) (Fig. 5*B*). A clear change in metabolic poise was observed upon SARS-CoV-2 infection in Calu-3 cells, where a majority of the significantly upregulated proteins belonged to glycolysis/gluconeogenesis and fructose and mannose metabolism, whereas most of the proteins of the TCA cycle were significantly downregulated (Fig. 5*B* and supplemental Fig. S6). However, this phenomenon was not observed in the other three cell culture models (supplemental Fig. S7). Only two of 177 proteins identified were significantly different in Caco-2 cells (ACSS1 and PFKFB4), and no differences were observed in Huh7 cells out of the four identified proteins in the three pathways mentioned previously (supplemental Fig. S6). This shows that Calu-3 cells, which are lung epithelial cells, have a distinct metabolic modulation caused by SARS-CoV-2 infection. Interestingly, although all the mitochondrial TCA cycle enzymes were downregulated, cytosolic enzymes, such as MDH1, IDH1, ACO1, and ACLY, that convert TCA cycle intermediates outside the mitochondria were upregulated in infected Calu-3 cells (Fig. 5*C*). This points toward dysfunctional mitochondria caused by COVID-19 infection. Alterations in mtDNA copy number in circulating blood cells can serve as a surrogate for mitochondrial dysfunction (34). Indeed, in our patient cohort, we observed a decreasing trend of the mtDNA copy numbers with the disease severity (Fig. 5*D*). In addition to changes in glucose and glutamate (Fig. 3*D*), we also observed a significant increase in metabolites, such as pyruvate, lactate, and α-ketoglutarate (more pronounced in mild patients) and decrease in citrate and aconitate in COVID-19 patients compared with HCs (supplemental Fig. S4). This indicated an impact of SARS-CoV-2 infection on glycolysis and glutaminolysis to meet biosynthetic and bioenergetic demands. In order to determine the requirement of glycolysis and glutaminolysis for optimal replication of SARS-CoV-2 in Calu-3 cells, we blocked these pathways using 2-DG (10 mM) and DON (200 μM), respectively (Fig. 5*E*). The concentrations were selected based on the cell cytotoxicity assays (Fig. 5*F*). Infectivity of SARS-CoV-2, quantified as relative *E-gene* levels in cell lysates, showed ∼50-fold decrease in 2-DG–treated cells and >100-fold decrease in DON-treated cells (Fig. 5*G*). This was also corroborated with virus production in the cell culture supernatant, quantified by viral *E-gene* levels that decreased by more than 2log10 RNA copies/ml in both 2-DG- and DON-treated cells compared with untreated cells (Fig. 5*H*). While several studies have shown the role of glycolysis on SARS-CoV-2 infection (25, 32), so far there is no direct evidence linking the role of glutaminolysis to replication and spread of SARS-CoV-2, and here, we show for the first time that both glutaminolysis and glycolysis can be essential for SARS-CoV-2 infection and progressive replication *in vitro* in the lung epithelial cell line.

## Discussion

In this study, we used metabolomics, proteomics, and immunophenotyping to observe the effect of SARS-CoV-2 infection on metabolic dysregulation in COVID-19 patients and reanalyzed our earlier *in vitro* infection data in four different cell models to find out potential host metabolic regulation during acute SARS-CoV-2 infection. Our study recognized the role of monocytes, especially in severe COVID-19 disease. As expected, COVID-19 patients presented a cytokine storm. Interestingly, IL-12 plasma levels were decreased in severe compared with mild COVID-19 patients. Among the carbohydrates, plasma mannose emerged as a biomarker for disease severity, but *in vitro* assays showed no effect of mannose on viral replication. Furthermore, host cellular response following SARS-CoV-2 infection identified a strong acute metabolic adaptation in the lung epithelial cells (Calu-3) by modulating central carbon metabolism and indicative of mitochondrial dysfunction that is also observed in severe COVID-19 patients. Glycolysis and glutaminolysis can be essential for virus replication, and metabolic perturbations of these processes can impede SARS-CoV-2 and could be an attractive antiviral strategy.

As reported, the cytokine storm syndrome is evident in COVID-19 patients (35). Several plasma proinflammatory cytokines including IL-6 were elevated in both mild and severe COVID-19 patients. In our study, though we observed higher levels of soluble IL-12 in mild patients, the severe patients showed IL-12 levels like HCs. Elevated plasma levels of IL-12 have also been described in hospitalized patients infected with other coronaviruses, such as SARS-CoV and Middle East respiratory syndrome coronavirus (36). However, to our knowledge, no associations of IL-12 levels with disease severity were reported in these infections. *In vitro* studies on IL-12 administration have shown enhanced host cellular responses that generally promote virus clearance and host recovery from infection (37). IL-12 also plays a critical role in viral immunity by activating the natural killer cells and promoting differentiation of Th1 CD4^+^ T cells. Based on the different levels of IL-12 in COVID-19 patients at varying disease stages, its role in disease severity needs further attention.

Both DNA and RNA viruses rewire host cell metabolism by altering central carbon metabolic pathways such as glycolysis, gluconeogenesis, PPP, TCA cycle, amino acid synthesis/degradation, and lipid synthesis. In our metabolomics data, we observed increased glucose, pyruvate, and lactate levels in the plasma of COVID-19 patients compared with HCs indicative of toxic metabolic dysregulation during acute phase of infection. Under aerobic and standard growth conditions, primary mammalian cells use glucose for generation of ATP. Alterations of the amino acid metabolism in COVID-19 patients were reported earlier as observed in our study (1, 3, 4, 21, 38, 39, 40). Off note, the amino acid metabolic pathways are dependent upon the central carbon metabolism, including glycolysis, TCA cycle, glutaminolysis, and urea cycle. Moreover, earlier studies including ours reported that SARS-CoV-2 modulates AKT/mTOR/HIF-1 pathways that is central regulator of the glycolysis and other central carbon metabolic pathways (7, 25, 39). Combining ours and other studies one can posit that the SARS-CoV-2 infection alters the key metabolic pathways of central carbon metabolism that are regulated by AKT/mTOR/HIF-1 signaling and may potentially define disease severity.

Viral infections including SARS-CoV-2 are known to enhance the glycolytic flux and increase the production of lactate from pyruvate (41, 42). Viruses can target glycolysis by regulating glucose transporters' expression, which is also vital for immune cell activation during host cellular response (43). Increased GLUT1 does not only result in higher glucose uptake but also gives rise to increased PPP intermediates that enhance nucleotide pool required for viral replication (6). GLUT1 transports glucose, mannose, glucosamine, and docosahexaenoic acid across the cell membrane (44). We observed a significant increase in surface expression (mean fluorescence intensity) of GLUT1 in CD8^+^ T cells and intermediate monocytes in COVID-19 severe patients. We also measured surface expression of xCT, a cystine/glutamate antiporter that exchanges glutamate for cystine essential for maintenance of redox balance. Furthermore, we observed a significant increase in surface expression of xCT in classical and intermediate monocytes in COVID-19 patients. To the best of our knowledge, the association of xCT expression in respiratory viral diseases has not been studied before, and our data for the first time highlight a potential role of expression of xCT in monocytes that needs further investigation. A growing body of evidence highlights the potential role and metabolic status of monocytes in COVID-19 disease severity (4, 31). A recent study also reported the potential role of GLUT1 in the disease progression of SARS-CoV-2 infection (45). Combining all the data, metabolite transporters, xCT and GLUT1, could play an essential role in disease severity. The specific changes of metabolic transporters were more prominent in monocytes, indicating that metabolic profile of the different monocytic subpopulations could contribute to mediating severity of the disease.

Plasma mannose emerges as a robust biomarker of disease severity that is in line with earlier studies from China and United States (3, 4) indicating the role of mannose in COVID-19 severity. Other metabolite biomarkers, like 6-oxopiperidine-2-carboxylate, hydantoin-5-propionate, 4-hydroxy phenylacetate, eicosanedioate, and 6-bromotryptophan, were not reported earlier. This could be because of patient heterogeneity, genetic background, diet, or the metabolomics method used. In addition to mannose, we also observed increased levels of MBL in both COVID-19 patients and convalescent patients compared with the COVID-19–negative individuals. However, no correlation between plasma MBL and mannose were observed in COVID-19, and these two can very well be independent factors. Monomeric mannose is a crucial element of N-linked glycosylation of proteins. Recent studies reported that plasma mannose levels were an indicator of glycogenolysis as well as glucose tolerance and associated with the future risk of developing chronic diseases, such as type 2 diabetes, cardiovascular diseases, and albuminuria (46). In our study cohort, the incidence of type 2 diabetes was low in all study groups and can therefore not explain the high plasma mannose levels in COVID-19 patients. This further strongly suggests that increased mannose is an effect of SARS-CoV-2 infection. However, considering recent research indicating a possibility of a bidirectional link between SARS-CoV-2 and diabetes, it is tempting to speculate that increased mannose has a role to play in new-onset diabetes after SARS-CoV-2 infection (47, 48).

C-type lectins, such as MBL, recognize carbohydrates, particularly on the surface of microorganisms leading to activation of the complement cascade and phagocytosis (23). Although N-linked mannose residues have been identified on SARS-CoV-2 spike protein, it is improbable that the elevated plasma mannose levels in the patients would be derived from the virus itself (49). The processing of endogenous glycoconjugates and their subsequent efflux from the cells are currently thought to be responsible for mannose levels in the blood and steady-state maintenance (50). Interestingly, a recent study by Heindel *et al.* (51) describes endogenous high mannose levels as a key mediator of influenza virus–induced pathogenesis and disease severity. High mannose is induced through unfolded protein response pathway, and the influenza virus–infected cells are recognized in a high mannose–dependent manner by MBL (51). Finally, authors state that MBL signaling contributes to disease severity through complement cascade activation and inflammatory response. High mannose and/or high MBL could thus dysregulate the immune system and lead to severe damage associated with disease severity (51). Activation of the complement is one of the features seen in COVID-19, as described in earlier studies (7, 52). In concordance with Heindel *et al.* as reported in influenza, we also noticed increased plasma MBL in COVID-19 and even in healthy convalescent controls compared with HC. This increase in MBL could potentially regulate the complement cascade.

To elucidate the effects of extracellular glucose and mannose in infection and replication of SARS-CoV-2, we established an *in vitro* infection set up with varying media concentrations of glucose and mannose postinfection. Virus production in the cell culture supernatant was unaffected by both glucose and mannose concentrations. However, high extracellular glucose decreased viral infectivity, measured as relative expression of viral *E-gene* in Calu-3 cells. This contradicts the earlier finding that an increase in glucose concentration aids in virus replication (25). However, it is to be noted, the observations by Codo *et al.* (25) were made in peripheral monocytes, whereas we performed our experiments in Calu-3 as we have reported cell-specific replication dynamics and immune modulation in SARS-CoV-2 (9).

In the untargeted quantitative proteomics of the Calu-3 infection model, most of the proteins from carbohydrate metabolism and PPP were upregulated, whereas most of the proteins of TCA cycle, oxidative phosphorylation, and fatty acid metabolism were downregulated in infected cells compared with the mock-infected controls (Fig. 5). Further delineation of the pathways indicated an inefficient mitochondrial metabolism as majority of the TCA cycle enzymes were downregulated in the infected cells. A significant downregulation of the TCA cycle and glycolytic pathways in COVID-19 patients compared with COVID-19 negative samples in plasma proteomics reported earlier corroborates our findings (39). This was in line with the decreased mtDNA copy numbers in severe COVID-19 patients (Fig. 5*F*) indicating a possible mitochondrial dysfunction as reported previously (31, 53).

The metabolism and concentration of sugars and amino acids, such as glucose, mannose, glutamine, and glutamate among others, play an important role in cellular metabolic homeostasis and are targeted by viruses for their replication (5). Recent studies have shown that elevated glycolysis favors SARS-CoV-2 infection and replication (25, 32). Glutaminolysis has been implicated as a carbon source for other human DNA and RNA viruses (6). Our data show for the first time that glutaminolysis is also crucial for SARS-CoV-2 infection and replication. The inhibition of glutaminolysis has a larger effect on viral replication and production compared with the inhibition of the glycolysis in lung cell model (Fig. 5, *F* and *G*). Glutaminolysis is a process of converting glutamine to TCA cycle intermediates and essential for biosynthesis of proteins, lipids, and nucleic acids. Some viruses (*e.g.*, herpes simplex virus 1, human cytomegalovirus, hepatitis C virus, etc) use glutamine as an anaplerotic substrate to replenish TCA cycle *via* generation of α-ketoglutarate (42, 54). Recently, researchers have proposed that the metabolic reprogramming of glutamine in SARS-CoV-2 can trigger pathogenesis. They further hypothesized that metabolic intervention of glutaminolysis could be an antiviral strategy for COVID-19 (54, 55). Although the exact underlying mechanism is unknown, our *in vitro* study shows that SARS-CoV-2 replication depends on both glycolysis and glutaminolysis.

In conclusion, our patient-based multiomics studies and *in vitro* analysis emphasizes the need to understand the host metabolic reprogramming because of acute SARS-CoV-2 infection. Among other factors, the role of carbohydrate and amino acid transporters, mainly in the monocytic–macrophage lineages, under the altered central carbon metabolism regulated by AKT/mTOR/HIF-1 signaling may potentially define disease severity. The metabolic alteration in glucose, mannose, lactate, pyruvate, and glutamate levels in severe COVID-19 cases need further clinical considerations. Changes in these metabolites might have a sustained effect on insulin resistance, type 2 diabetes, neurocognitive impairments, and multiorgan failure, which is already reported in COVID-19 infection.

## Data availability

All the data generated or analyzed during this study are included in this published article and/or the supplemental data. Created datasets and code are publicly available. The metabolomics data can be obtained from dx.doi.org/10.6084/m9.figshare.13336862. Untargeted quantitative proteomics data can be obtained from the ProteomeXchange Consortium *via* the PRIDE partner repository with the dataset identifier PXD023760. All the codes are available at github: https://github.com/neogilab/COVIDOMICS.

## Supplemental data

This article contains supplemental data.

## Conflict of interest

The authors declare no competing interests.
