# Supplementary material for: Metabolic Perturbation Associated With COVID-19 Disease Severity and SARS-CoV-2 Replication
Source: Mol Cell Proteomics. 2021 Oct 5;20:100159. doi: 10.1016/j.mcpro.2021.100159 (PMC8490130; doi:10.1016/j.mcpro.2021.100159)
Supplement: Supplemental Tables S1–S3 and Figures S1–S7 [file mmc1.pdf]

Supplementary Materials for

Metabolic perturbation associated with COVID-19 disease severity and SARS-CoV-2 replication

Authors: Shuba Krishnan[1], Hampus Nordqvist[2], Anoop T. Ambikan[1], Soham Gupta[1], Maike Sperk[1], Sara Svensson-Akusjärvi[1], Flora Mikaeloff[1], Rui Benfeitas[3], Elisa Saccon[1], Sivasankaran Munusamy Ponnan[4], Jimmy Esneider Rodriguez[5], Negin Nikouyan[1], Amani Odeh[6], Gustaf Ahlén[1], Muhammad Asghar[6], Matti Sällberg[1], Jan Vesterbacka[7], Piotr Nowak[7,8], Ákos Végvári[5], Anders Sönnerborg[1,6], Carl Johan Treutiger[2,7], Ujjwal Neogi[1*].

*Correspondence to: Ujjwal Neogi (ujjwal.neogi@ki.se)

This PDF file includes:

Tables S1 to S3
Figures. S1 to S7

Table S1.

List of reagents, kits and antibodies

**Antibodies**		
CD4-BUV395-SK3	BD Biosciences	563552
CD8-APC-RPA-T8	Biolegend	301014
CD14-BV510-M5E2	Biolegend	301842
CD3-BV711-OKT3	Biolegend	317328
CD16-BV786-3G8 (RUO)	BD Biosciences	563690
GLUT1-FITC-# 202915	R&D Systems	FAB1418F
xCT-AF594	Novus Biologicals	NB300-318AF594
CD8-PerCP-HIT8a	Biolegend	300922
CD3-FITC-OKT3	Biolegend	317306
Anti-Human IgG Peroxidase	Sigma-Aldrich	A0170
SARS-CoV-2 N protein	Varnaitė et al., 2020	GenScript
Tetramethylbenzidine	Sigma-Aldrich	T0440
**Critical Commercial Assays**		
Olink Immuno-Oncology	Olink Technology, Sweden	Immuno-Oncology Panel
Global Metabolomics (HD4)	Metabolon. Inc., US	HD4
Human MBL Quantikine ELISA Kit	R&D systems	DMBL00
Anti-Mouse Ig, κ/Negative Control Compensation Particles Set	BD Biosciences	552843
AbC™ Total Antibody Compensation Bead Kit	Invitrogen	A10513
ArC™ Amine Reactive Compensation Bead Kit	Invitrogen	A10346
DMEM-high glucose	Sigma-Aldrich, USA	D6429-500ml
Bio-rad DC protein assay kit	Bio-Rad Laboratories, USA	#5000116
TaqMan Fast Virus 1-Step Master Mix	ThermoFisher Scientific	4444434
Direct-zol™ RNA Miniprep Kit	Zymo Research	R2051
Tempus™ Blood RNA Tubes	Applied Biosystems	4342792
Tempus™ Spin RNA Isolation Kit	Invitrogen	4380204
PrimeDirect™ Probe RT-qPCR Mix	TaKaRa, Japan	RR600B
KAPA SYBR Fast qPCR kit	Roche	KK4602
TMTpro 16plex Label Reagent set	ThermoFisher Scientific	A44520
Absolute Human Telomere Length and Mitochondrial DNA Copy Number Dual Quantification qPCR Assay Kit	ScienCell Research Laboratories	#8958
QuantiNova SYBR® Green PCR Kit	Qiagen	208054
alamarBlue™ Cell Viability Reagent	Invitrogen	DAL1025

Table S2.
List of primer and probe sequences

E_Sarbeco_F1-5'-ACAGGTACGTTAATAGTTAATAGCGT-3'	WHO
E_Sarbeco_R2-5'-ATATTGCAGCAGTACGCACACA-3'	WHO
E_Sarbeco_Probe-5'-[FAM]-ACACTAGCCATCCTTACTGCGCTTCG-[BBQ650]-3'	WHO
RNAseP-F-5'-AGATTTGGACCTGCGAGCG-3'	CDC
RNAseP-R-5'-GAGCGGCTGTCTCCACAAGT-3'	CDC
RNAseP-Probe-5'-[FAM]-TTCTGACCTGAAGGCTCTGCGCG-[BHQ1]-3'	CDC

Table S3.

Clinical features of study population

Parameter	HC	Hospitalised-Mild	Hospitalised Severe	P values*	P values**
number	31	29	12		
Age, years; median (IQR)	48 (46-55)	57 (44-63)	57 (52-69)	0.0277	0.2831
Gender, Male, n (%)	24 (77%)	23 (79%)	11 (91%)	0.557	0.6514
BMI, Median (IQR)	24 (21-25)	29 (25-31)	28 (25-34)	0.0016	0.8622
Sample collection post hospitalisation, days, median (IQR)	-	2 (1-3)	3 (2-4)		0.1170
Comorbidities, yes (%)	-	13 (45%)	8 (66%)		0.3058
Obesity, yes (%)		2 (7%)	1 (8%)		1
Type 2 Diabetes, yes (%)		2 (7%)	1 (8%)		1
Hypertension, yes (%)		7 (24%)	4 (33%)		0.7011
Asthma, yes (%)		4 (14%)	3 (25%)		0.3978

*within the groups, **between hospitalized mild and severe

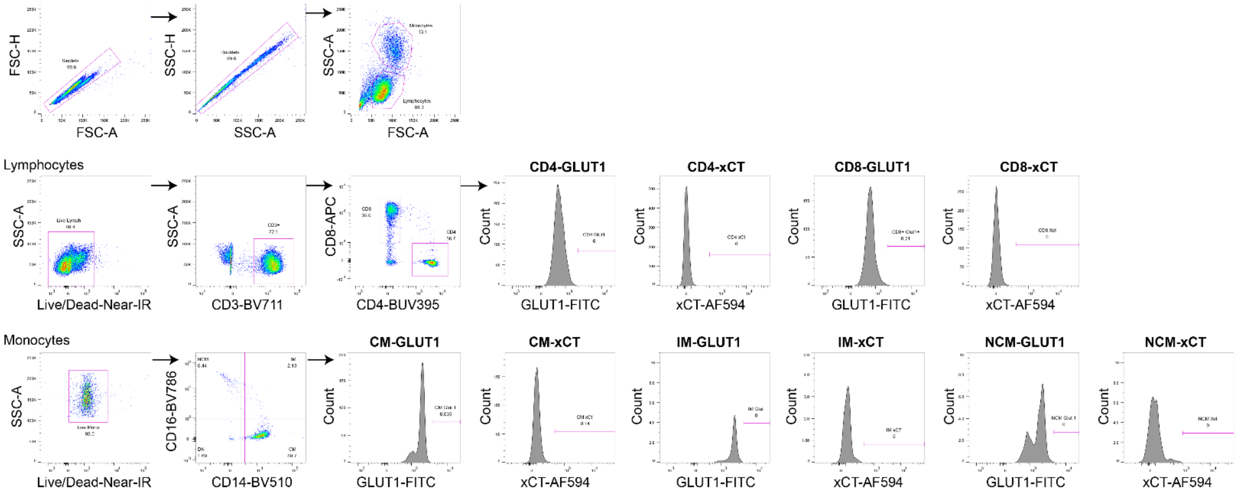

Figure S1: Gating strategy of flow cytometry data.

Pt ID	Plate no.	Mean (1:200)	Mean (1:400)	Mean (1:800)	Mean (1:1600)	Antibody status
HC-03	1	2,97	2,43	1,470	0,840	POS
HC-10	1	3,00	2,70	1,768	0,995	POS
HC-12	2	3,00	2,32	1,370	0,756	POS
HC-14	2	2,70	1,65	0,937	0,526	POS
HC-15	2	3,00	3,00	3,000	3,000	POS
HC-18	2	2,69	1,52	0,859	0,427	POS
HC-23	3	3,00	2,82	1,825	1,120	POS
HC-26	3	2,87	2,00	1,119	0,581	POS
HC-13	2	0,62	0,36	0,178	0,098	POS
HC-32	3	0,19	0,10	0,063	0,043	POS
HC-21	3	0,16	0,09	0,051	0,042	NEG
HC-06	1	0,16	0,08	0,045	0,029	NEG
HC-17	2	0,15	0,09	0,053	0,038	NEG
HC-31	3	0,14	0,07	0,046	0,037	NEG
HC-22	3	0,13	0,07	0,043	0,036	NEG
HC-24	3	0,12	0,07	0,045	0,034	NEG
HC-25	3	0,14	0,06	0,042	0,030	NEG
HC-01	1	0,10	0,05	0,037	0,024	NEG
HC-02	1	0,06	0,03	0,023	0,019	NEG
HC-04	1	0,09	0,05	0,033	0,025	NEG
HC-05	1	0,03	0,02	0,015	0,020	NEG
HC-07	1	0,05	0,03	0,019	0,014	NEG
HC-08	1	0,08	0,04	0,028	0,021	NEG
HC-09	1	0,07	0,04	0,026	0,020	NEG
HC-11	2	0,08	0,05	0,032	0,124	NEG
HC-16	2	0,08	0,05	0,032	0,023	NEG
HC-19	2	0,05	0,03	0,026	0,019	NEG
HC-20	2	0,06	0,04	0,028	0,023	NEG
HC-27	3	0,10	0,07	0,042	0,033	NEG
HC-28	3	0,07	0,04	0,032	0,027	NEG
HC-29	3	0,07	0,04	0,029	0,024	NEG
Considered Ab+ ≥		0,17	0,11	0,08	0,07	

Figure. S2. The IgG Ab showed 10 of the HC were were CoV-2 Ab-positive.

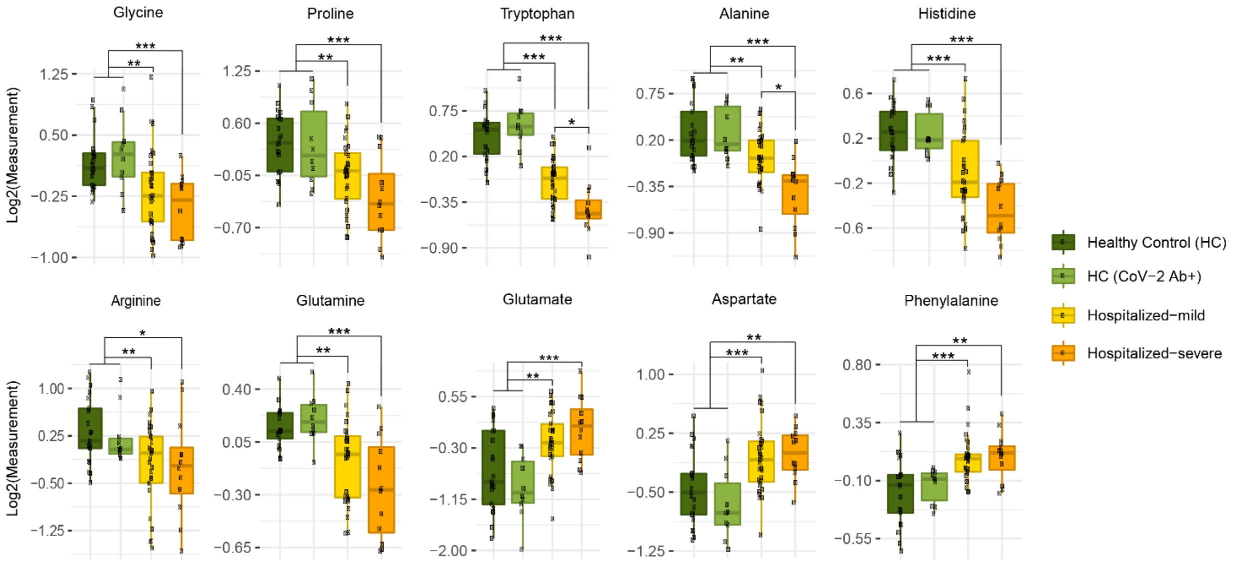

Figure. S3. Metabolite profile of amino acids altered in COVID-19 patients. Line within box plots represents median values, *p<0.05, **p<0.01, ***p<0.001.

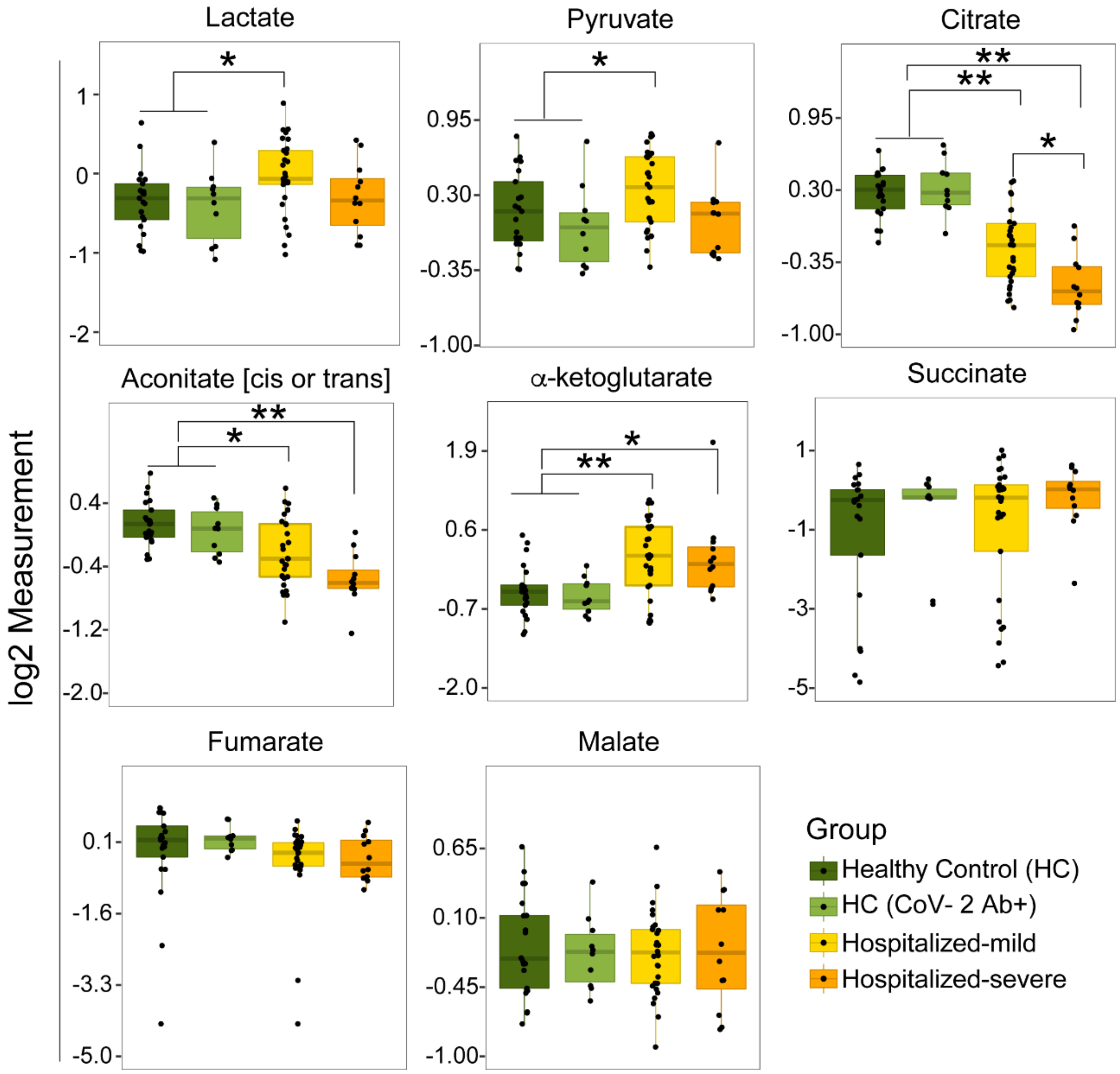

Figure. S4. Levels of metabolites related to glycolysis/gluconeogenesis and fructose and mannose metabolism and the TCA cycle.

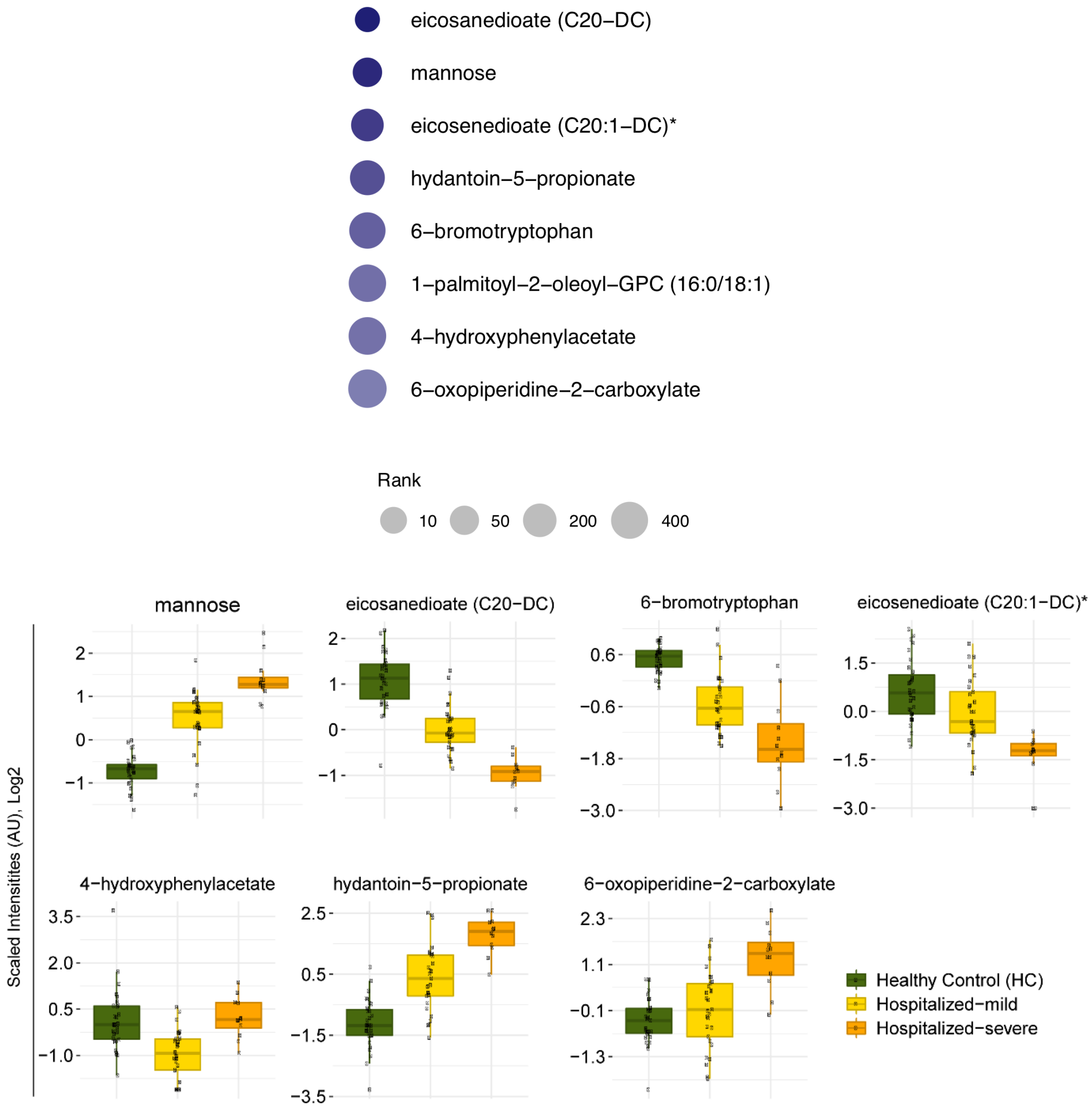

Figure. S5. Biomarker of the COVID-19 severity identified by MUVR. Size of the bubble indicates rank. Box plot of the biomarkers indicating the level in HCs and COVID-19 patients.

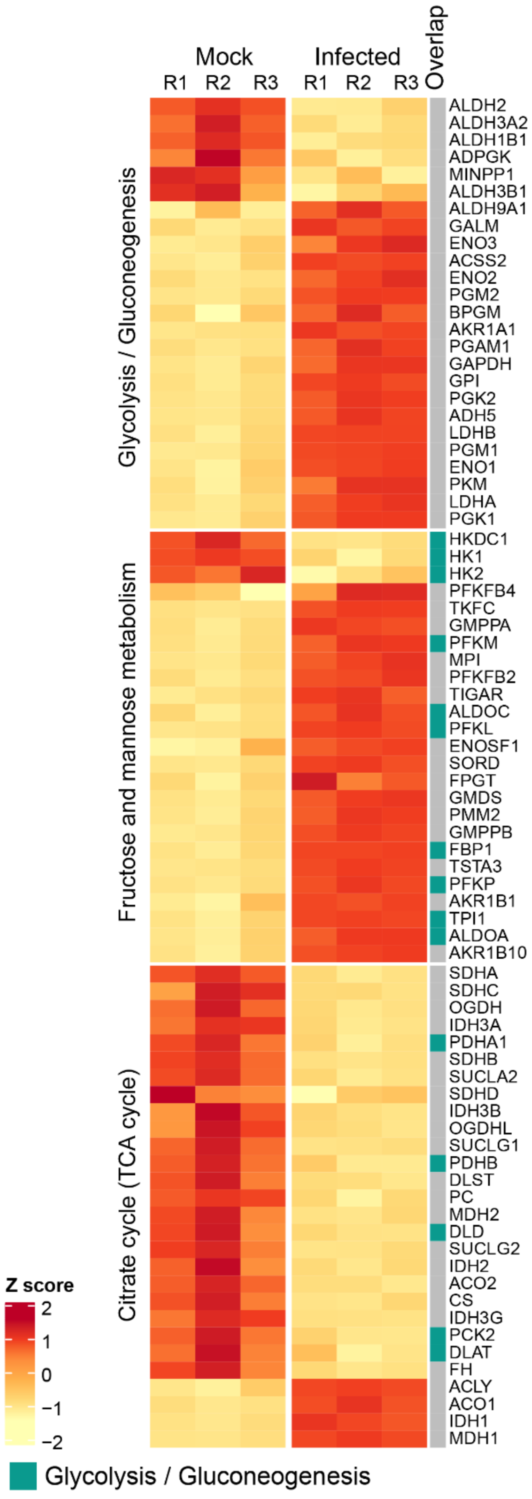

Figure S6: Differential protein abundance in Calu-3 cells following SARS-CoV-2 infection after 24 hrs. The analysis was restricted to glycolysis/gluconeogenesis, fructose and mannose metabolism and TCA cycle.

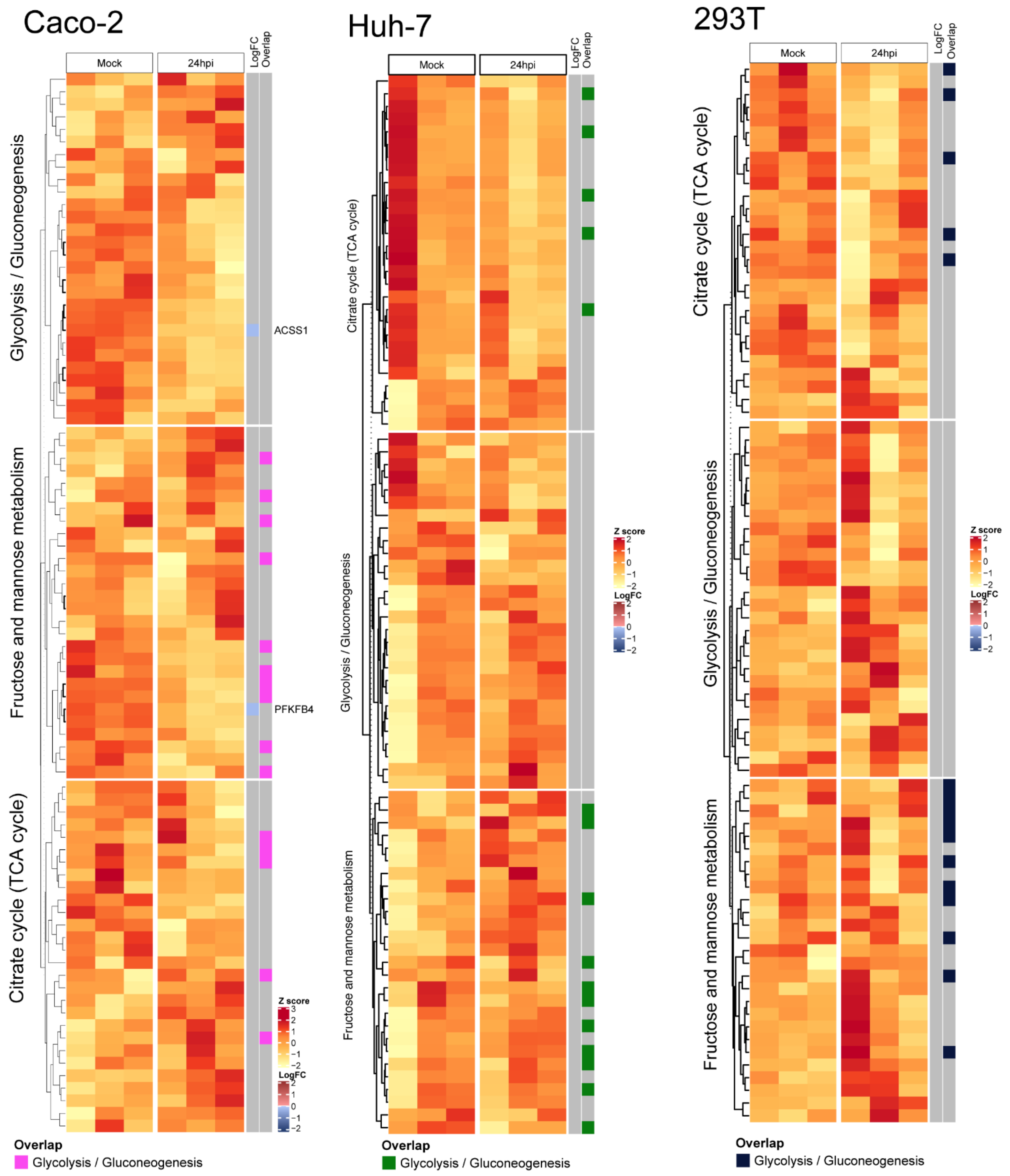

Figure S7: Differential protein abundance in Caco-2, Huh7 and 293FT cells following SARS-CoV-2 infection after 24 hrs. The analysis was restricted to glycolysis/gluconeogenesis, fructose and mannose metabolism and TCA cycle.
